# The first South American sandownid turtle from the Lower Cretaceous of Colombia

**DOI:** 10.7717/peerj.1431

**Published:** 2015-12-15

**Authors:** Edwin Cadena

**Affiliations:** Centro de Investigaciones Paleontológicas, Villa de Leyva, Colombia; Alexander von Humboldt Foundation, Senckenberg Naturmuseum, Frankfurt am Main, Germany

**Keywords:** Testudines, Lower Cretaceous, South America, Paleobiogeography, Colombia, Sandownidae, Durophagous, Villa de Leyva, Global phylogeny

## Abstract

Sandownids are a group of Early Cretaceous-Paleocene turtles that for several decades have been only known by cranial and very fragmentary postcranial elements. Here I report and describe the most complete sandownid turtle known so far, including articulated skull, lower jaw and postcranial elements, from the Early Cretaceous (upper Barremian-lower Aptian, >120 Ma), Paja Formation, Villa de Leyva town, Colombia. The new Colombian sandownid is defined here as *Leyvachelys cipadi* new genus, new species and because of its almost identical skull morphology with a previously reported turtle from the Glen Rose Formation, Texas, USA, both are grouped in a single and officially (ICNZ rules) defined taxon. Phylogenetic analysis including *L. cipadi* supports once again the monophyly of Sandownidae, as belonging to the large and recently redefined Pan-Chelonioidea clade. The morphology of *L. cipadi* indicates that sandownids were not open marine turtles, but instead littoral to shallow marine durophagous dwellers. *Leyvachelys cipadi* not only constitutes the first record of sandowinds in South America, but also the earliest global record for the group.

## Introduction

Pan-cryptodires (sensu Joyce, Parham & Gauthier, 2004) represent globally distributed and highly diverse turtles with a wide spectrum of environmental adaptations (coastal-littoral, freshwater, and terrestrial) and body-plan (e.g., Pérez-García, 2012; Anquetin, Püntener & Billon-Bruyat, 2014; Jansen & Klein, 2014; Rabi et al., 2014; Tong et al., 2014; Anquetin, Püntener & Billon-Bruyat, 2015; Zhou & Rabi, 2015). Among pan-cryptodires, Sandownidae is a very particular group of turtles, formed by four taxa, including: *Sandownia harrisi* (Meylan et al., 2000) from the Aptian of the Isle of Wight, England; *Angolachelys mbaxi* (Mateus et al., 2009) from the Turonian of Angola; an unnamed turtle (“the Glen Rose turtle”) from the Albian Glen Rose Formation of Texas (Barck, 1992; Vineyard, 2009), and *Brachyopsemys tingitana* (Tong & Meylan, 2013) from the Early Paleocene (Danian) of Morocco. The phylogenetic position and closer affinities of sandownids inside pan-Cryptodira (sensu Joyce, 2007) are still highly controversial. Initially, sandownids, particularly *S. harrisi* was considered as the most basal representative of Trionychia (see Meylan et al., 2000). In a global phylogenetical study of turtles performed by Joyce (2007) *S. harrisi* was considered as a rouge taxon lacking firm affinities, but potentially belonging to “*Santanachelys* Node, Clade 20”, and less closer to Trionychia clade. With the description of *A. mbaxi*, Mateus et al. (2009) included the three sandownids described by then in the matrix of Joyce (2007), finding them as monophyletic group, with the inclusion of the Jurassic turtle *Solnhofia parsoni* (Gaffney, 1975), defining a new clade called Angolachelonia. With the discovery and description of *B. tingitana* by Tong & Meylan (2013), all sandownids (excluding “the Glen Rose turtle”) were included in a phylogenetic study, placing it as the sister group of a clade including the extant marine turtles Dermochelyidae, Cheloniidae and some other fossil marine forms including *Santanachelys gaffneyii* (Hirayama, 1998) and *Tasbacka ouledabdounensis* (Tong & Hirayama, 2002). Another important finding of the phylogenetic analysis of Tong & Meylan (2013) was the exclusion of *So. parsoni* from Sandownidae, finding it more basal inside Eucryptodira. One of the main reasons for all these controversial results concerning the phylogenetic position of sandownids and their composition is the lack of complete articulated postcranial material. Anatomical and paleoecological interpretations of sandownids have suggested that they were durophagous and bottom-dwelling turtles, inhabiting near-shore shallow marine environments (Vineyard, 2009; Tong & Meylan, 2013); however, whether they were adapted to open marine conditions as other lineages of turtles, (see Cadena & Parham, 2015, for discussion about marine lineages of turtles), is still poorly supported. This is due to the lack of limbs and any other postcranial found associated to skull material.

**Figure 1:**
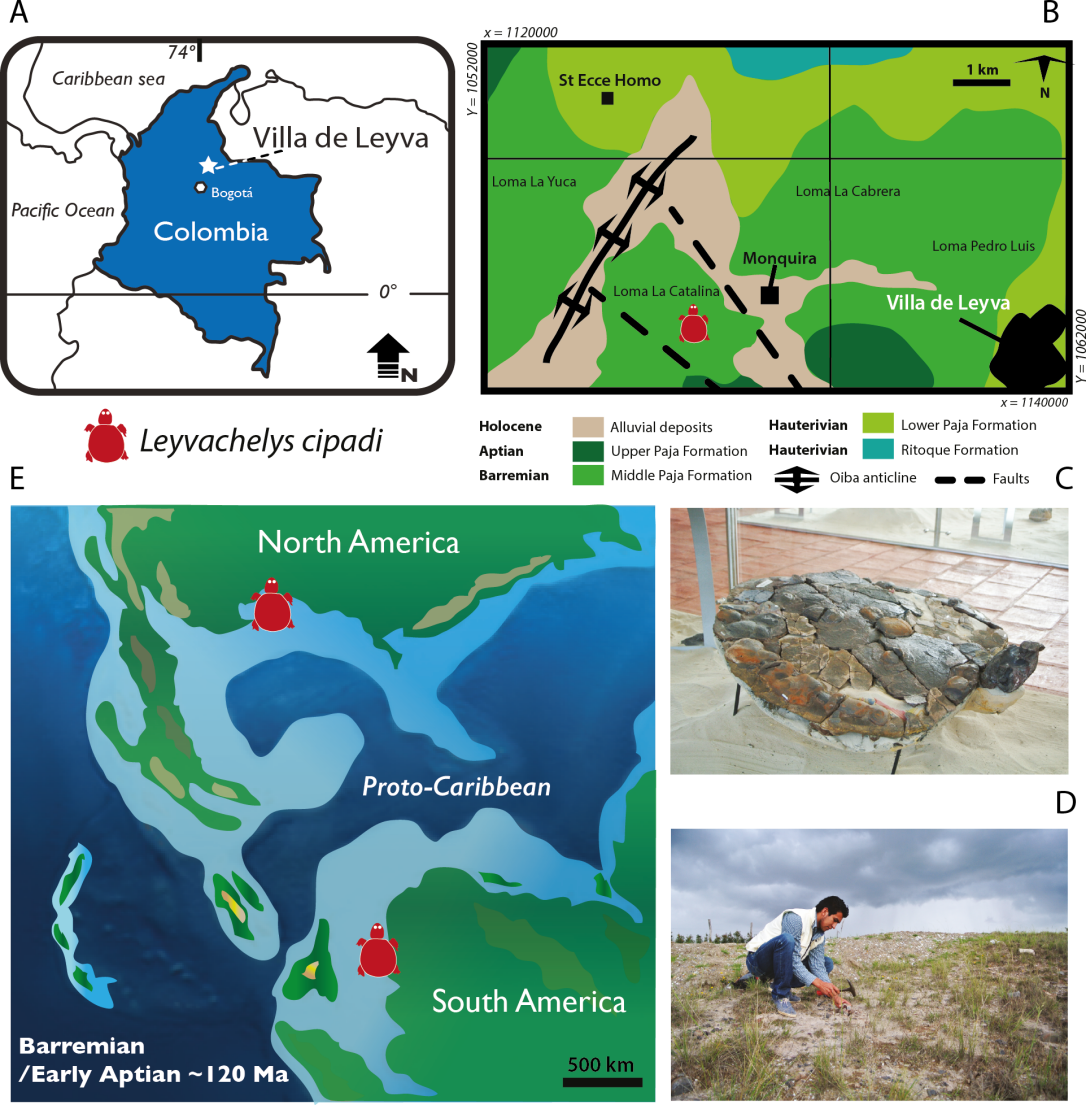
*Leyvachelys cipadi* FCG-CBP-71 location, geological and paleogeographical context. (A) map of Colombia showing the geographical location of Villa de Leyva town. (B) geologic map of the area where FCG-CBP-71 was found, redrawn from Hoedemaeker (2004). (C) *L.cipadi* FCG-CBP-71 exhibited at the Centro de Investigaciones Paleontológicas (CIP). (D) Fredy Parra who discovered *L. cipadi* FCG-CBP-71 at the Loma La Catalina. (E) paleotectonic reconstruction for the Barremian-Aptian of the proto-Caribbean region, redrawn from Blakey (2011) showing the occurrence of *L. cipadi* in Texas and Colombia.

In 2009, members of the Centro de Investigaciones Paleontológicas de Villa de Leyva (CIP) (Center for Paleontological Investigations) uncovered a nearly complete fossil turtle, at the Loma La Catalina, Villa de Leyva, Colombia (Figs. 1A and 1B). The fossil was found in a layer of calcareous claystone with abundant occurrences of ferruginous-calcareous nodules and concretions, belonging to the middle segment of the Paja Formation called “Arcillolitas abigarradas Member,” upper Barremian-lower Aptian, >120 Ma in age, based on ammonoids (Hoedemaeker, 2004, and references therein). Here, I describe the new fossil turtle from Villa de Leyva, which represents the most complete and earliest record for sandownids, and the first record of this group of turtles in South America. I discuss the phylogenetical, paleoecological, and paleobiogeographical implications of this new genus and species. At the same time, this is the second group of fossil turtles reported for the Paja Formation in Villa de Leyva. The first one corresponds to a protostegid, recently described as the potentially oldest marine turtle *Desmatochelys padillai* Cadena & Parham (2015).

## Materials and Methods

The fossil specimen described here was prepared using sulfamic acid following the protocol described by Padilla (2011), as well as mechanical tools, specially to remove hard nodules. All preparation was performed at the CIP. It is important to point out, that although the specimen is relatively complete, the preservation is not optimal, in particular for the observation of sutures between bones or sulci between scutes.

The electronic version of this article in Portable Document Format (PDF) will represent a published work according to the International Commission on Zoological Nomenclature (ICZN), and hence the new names contained in the electronic version are effectively published under that Code from the electronic edition alone. This published work and the nomenclatural acts it contains have been registered in ZooBank, the online registration system for the ICZN. The ZooBank LSIDs (Life Science Identifiers) can be resolved and the associated information viewed through any standard web browser by appending the LSID to the prefix “http://zoobank.org/”. The LSID for this publication is: (urn:lsid:zoobank.org:act:F79B04E9-5976-4B44-8A7F-28C6073ACF31). The online version of this work is archived and available from the following digital repositories: PeerJ, PubMed Central and CLOCKSS.

The new sandownid taxon described here was added to a recent updated global turtle character-taxon matrix (Cadena & Parham, 2015), including 256 characters, with the addition of one character for the pterygoid posteromedial wing (Character 257), as well as some score modifications for some taxa (see Supplemental Information 1 and Supplemental Information 2 (Nexus file global matrix)). A phylogenetic analyses was run in TNT Version 1.1 (Goloboff, Farris & Nixon, 2008) using the following settings: outgroup (*Odontochelys semitestacea*), all characters were equally weighted, 38 characters were considered additive (ordered of PAUP) (7, 17, 22, 42, 47, 50, 53, 55, 57, 63, 66, 67, 72, 75, 85, 94, 99, 111, 115, 121, 128, 131, 137, 141, 164, 167, 182, 184, 204, 216, 218, 222, 237, 249, 250, 251, 255, and 257), collapsing rules TBR (branch swapping) used. The first analysis included all characters and taxa using traditional search (keeping all settings by default). One of the trees obtained from this first run was unlocked and edited to match the backbone constraint tree topology for the extant OTUs following the most comprehensive molecular analysis of turtle relationships (Crawford et al., 2015) as was recently applied by Cadena & Parham (2015). The analysis was run again using traditional search analysis, this time with the defined constraint (selected groups or OTUs), enforce constraint, collapse trees after search, considering some taxa as floaters (see Supplemental Information 1) and additive characters activated. The most parsimonious trees (MPT) was repeated until the MPTs were hit 30 times during each replicate as indicated in Rabi et al. (2013). A second analysis was run, this time just including the taxa used in Cadena & Parham (2015) plus the four sandownids. All settings were as in the first analysis (constraint, traditional search, additive floaters and TBR collapsing rule), however this time two more characters were treated as additive (characters 72 and 223). A strict consensus was obtained from each of these two runs. Bootstrap support values and Bremer decay indices were obtained for the second run (prefer phylogenetic hypothesis presented on here).

## Systematic Paleontology

**Table:** 

**TESTUDINES** Batsch, 1788
**PAN-CRYPTODIRA** Cope, 1868
**SANDOWNIDAE** sensu Tong & Meylan, 2013

***Leyvachelys* gen. nov.**

*Diagnosis*. As for type species (below).

*Etymology*. Combining ‘*Leyva*’ (from Villa de Leyva, town of where the discovery took place) and ‘*chelys*’ (Greek, turtle).

***Leyvachelys cipadi* sp. nov.**

(Figs. 2–4)

**Figure 2:**
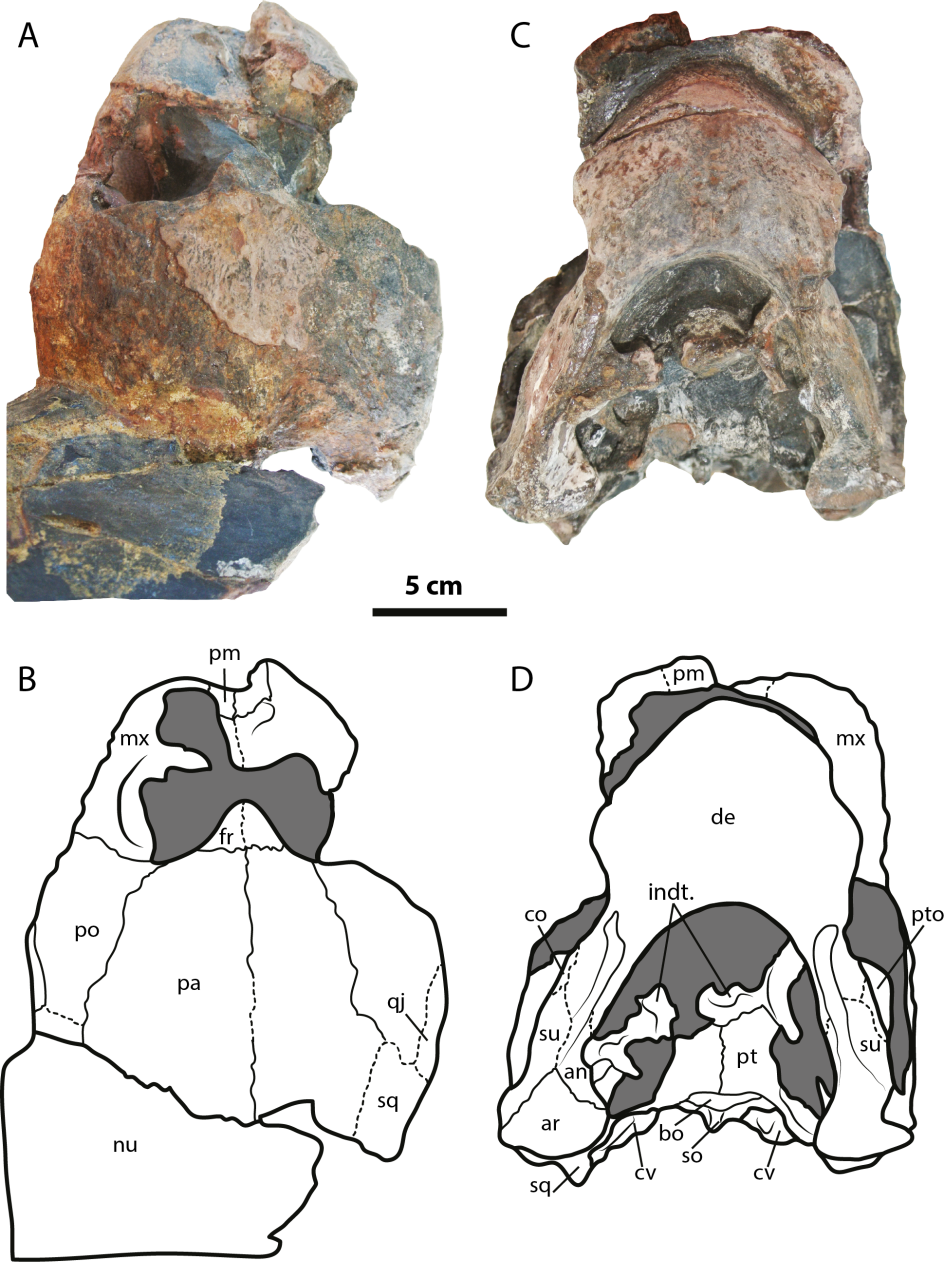
*Leyvachelys cipadi* FCG-CBP-71 skull and lower jaw. (A–B) dorsal view. (C–D) ventral view. Abbreviations: an, angular; ar, articular; bo, basioccipital; co, coronoid; cv, cervical vertebra; de, dentary; fr, frontal; indt, indeterminate bone fragments; mx, maxilla; nu, nuchal; pa, parietal; pm, premaxilla; po, postorbital; pt, pterygoid; pto, processus trochlearis oticum; qj, quadratojugal; sq, squamosal; so, supraoccipital; su, surangular. Dark grey areas indicate rock matrix.

**Figure 3:**
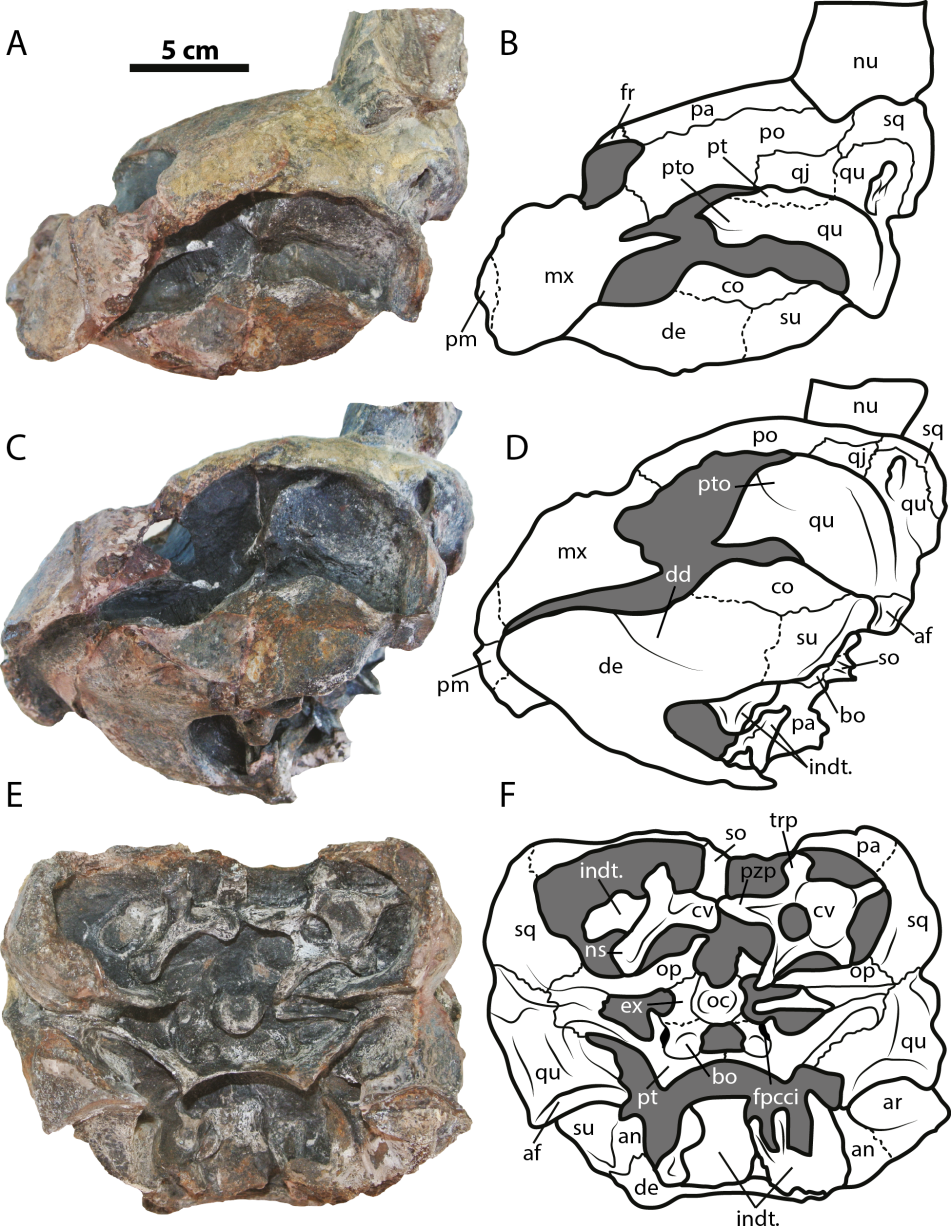
*Leyvachelys cipadi* FCG-CBP-71 skull and lower jaw. (A–B) left lateral view. (C–D) left ventrolateral view. (E–F) posterior view. Abbreviations: same as for Fig. 2, and: af, articular facet; dd, deep depression; ex, exoccipital; fpcci, foramen posterius canalis carotici interni; np, neural spine; pzp; prezygapophysis; op, opisthotic; qu, quadrate; trp, transverse process of cervical. Dark grey areas indicate rock matrix.

**Figure 4:**
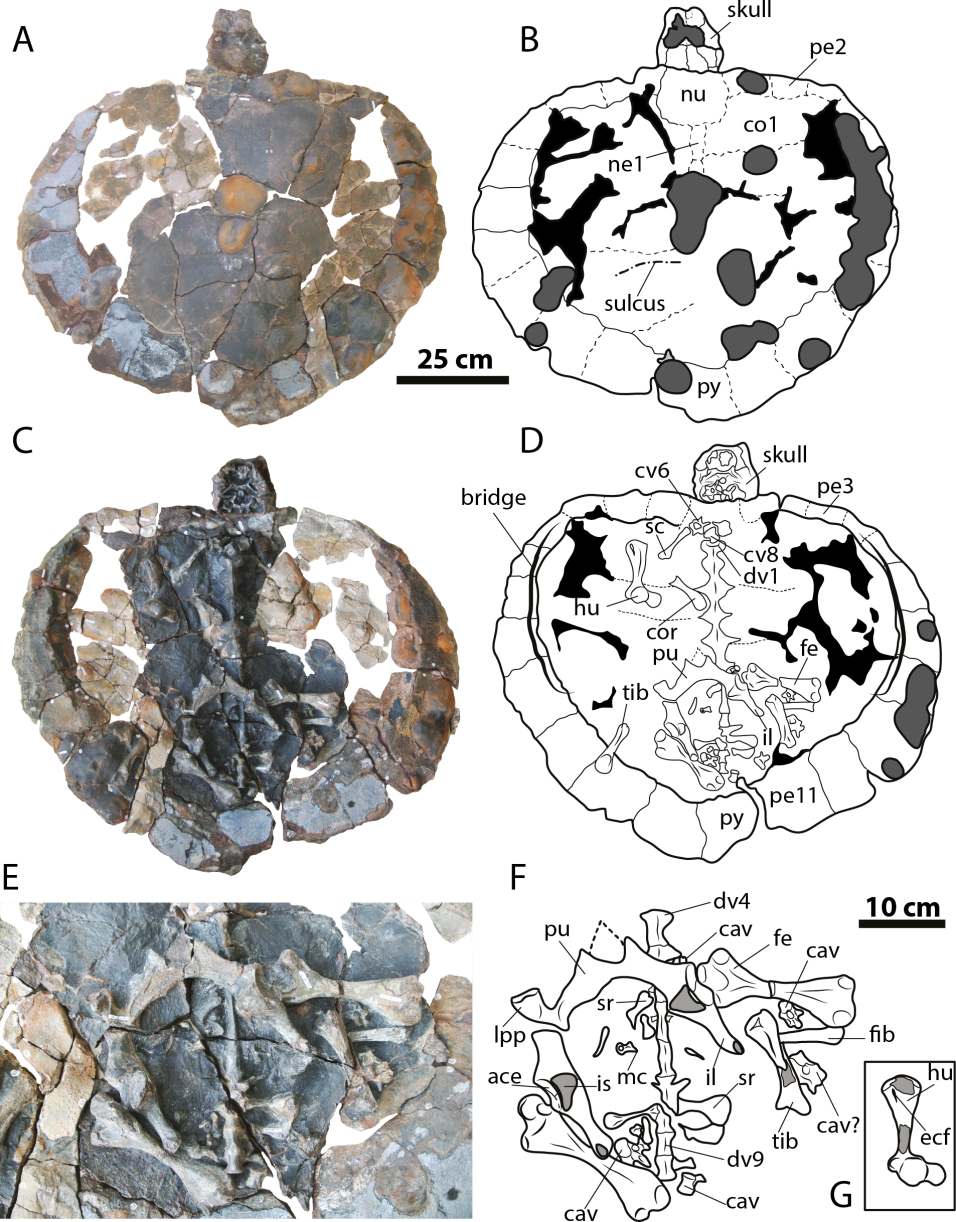
*Leyvachelys cipadi* FCG-CBP-71 whole specimen, skull, lower jaw and postcrania. (A–B) dorsal view. (C–D) ventral view. (E–F) pelvic and hindlimb elements, with the addition of the humerus in (G). Abbreviations: ace, acetabulum; cav, caudal vertebra, co, costal bone; cor, coracoid; cv, cervical vertebra; dv, dorsal vertebra; ecf, ectepicondylar foramen; fe, femur; fib, fibula; hu, humerus; il, ilium; is, ischium; lpp, lateral pubic process; mc, metacarpal; ne, neural bone; nu, nuchal; pe, peripheral; pu, pubis; py, pygal; sc, scapula; sr, sacral rib; tib, tibia. Dark grey areas indicate rock matrix, particularly nodules in C and D, and indicate bone in transversal cut in G. Black areas indicate missing bone.

*Etymology*. ‘*cipadi*’ (dedicated to the CIP, Centro de Investigaciones Paleontológicas)

*Holotype*. FCG-CBP-71 (housed at the CIP, Villa de Leyva, Colombia, Fig. 1C), articulated skull and lower jaw, nearly complete carapace, three cervical vertebrae, right humerus and coracoid, both femora, tibiae, and pelvic girdle, and two caudal vertebrae. For specimen measurements see Table 1.

**Table 1:** Measurements of *Leyvachelys cipadi* FCG-CBP-71 specimen as preserved.

	Value (Cm)
Skull	
Maximum length	15.6
Maximum width	15.1
Maximum height	12
Lower Jaw	
Maximum length	14.3
Maximum width	12.4
Carapace	
Maximum length	88
Maximum width	108
Right humerus	
Maximum length	17
Right Femur	
Maximum length	20.2
Left Femur	
Mximum length	20

**Figure 5:**
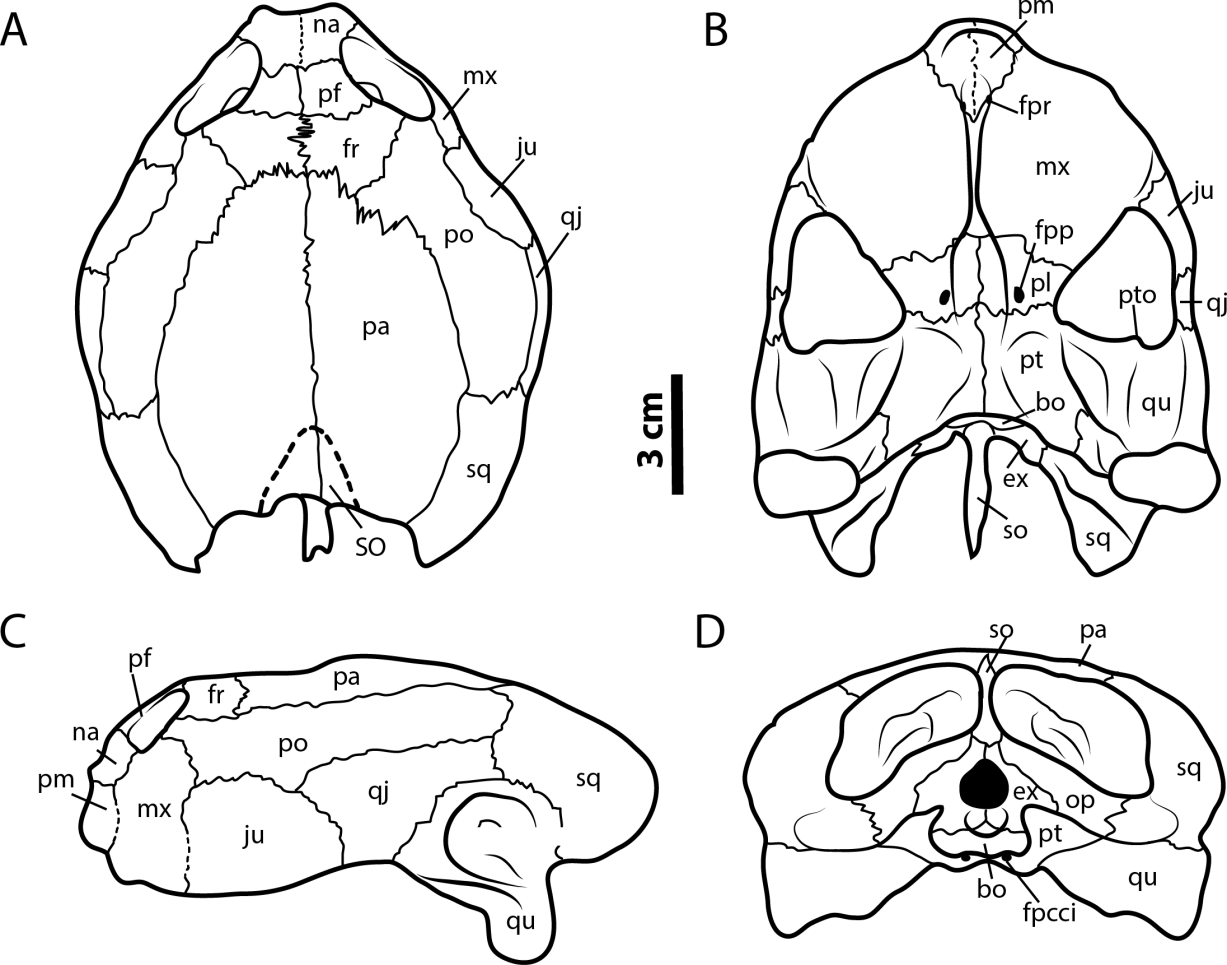
*Leyvachelys cipadi* FWMSH 93B-17 specimen. Redrawn from Vineyard (2009). (A) dorsal view, (B) ventral view, (C) left lateral view, (D) posterior view. Abbreviations: bo, basioccipital; ex, exoccipital; fr, frontal; fpcci, foramen posterius canalis carotici interni; fpp, foramen preapalatinum; fpr, foramen premaxillare; ju, jugal; mx, maxilla; na, nasal; pa, parietal; pm, premaxilla; pl, palatine; po, postorbital; pr, prefrontal; pt, pterygoid; pto, processus trochlearis oticum; qj, quadratojugal; sq, squamosal; so, supraoccipital; SO, supraoccipital scute; op, opisthotic; qu, quadrate.

*Referred specimens*. Six skulls previously described by *Vineyard (2009)* from Texas, USA (see remarks): SMU (Shuler Museum of Paleontology, Southern Methodist University) 75355, 72852, 74982, 74983, 75327; and FWMSH (Fort Worth Museum of Science and History) 93B-17 (Fig. 5).

*Occurrence and range*. Early Cretaceous (Barremian-lower Aptian, >120 Ma) “Arcillolitas abigarradas Member,” the Paja Formation, Loma La Catalina, Villa de Leyva, Colombia (Fig. 1D). Early Cretaceous (Albian), Glen Rose Formation, Texas, USA.

*Diagnosis. Leyvachelys cipadi* sp. nov. differs from all other sandownids by a premaxillary snout well defined with a straight anterior margin seen in ventral view of the skull; shallow and oval- shaped vacuity at the premaxillae-vomer sutural contact, allowing the ventral exposure of the foramina praepalatinum; narrow trough along the vomer length; laterally expanded palatine preventing a contact between the pterygoid and the maxilla or the jugal or both; narrow-to-broad shape of the quadratojugal from its cheek margin to its dorsal contact with the postorbital; a very long contact between the maxilla and the postorbital; jugal lacks of contribution to secondary palate. As all other sandownids, *L. cipadi* has a massive roof skull, including a parietal-squamosal and postorbital-squamosal contacts, with very broad and laterally expanded secondary palate; pterygoids partly or completely covering or surrounding basisphenoid, specially in adults; orbits placed anteriorly and facing forward; prominent and elongate processus trochlearis oticum extending anteriorly into the fossa temporalis inferior; occipital condyle positioned anterior or at the same level of the mandibular condyle; very long symphysis of dentary; a very tall coronoid; posterolateral border of the triturating surface bears a small ridge which delimits a relatively deep depression posteromedial to it, anterolateral to the processus coronoideus. *Leyvachelys cipadi* shares with *Brachyopsemys tingitana* a very wide anterior palatal region of the skull, with a very convex lateral margin of maxillae, wider than in *Sandownia harrisi*, *Angolachelys mbaxi*, and the Jurassic *Solnhofia parsonsi*. It shares with the other sandownids, except *B. tingitana* the absence of palatal pits, absence of prefrontal-jugal contact, presence of vomer-premaxillae contact, and a closed incisura columellae auris. *Leyvachelys cipadi* and the other sandownids, share a pterygoid posteromedial wing completely covering the basisphenoid and the most anterior portion of basioccipital, slightly less advanced in *S. harrisi*. It shares with *S. harrisi* premaxillae bones not completely fused medially. As in all other sandownids, except *Angolachelys mbaxi* (jaw unknown) the dentary has a very long symphysis and tall posteriorly hooked coronoid process. *Leyvachelys cipadi* shares with *B. tingitana*, a deep concave depression of the dentary and coronoid, located anterolateral to the processus coronoideus. *Leyvachelys cipadi* differs from *B. tingitana* and *S. harrisi* in having a dentary with a flat ventral surface, without an anteromedial deep sulcus.

*Remarks*. Turtle skulls from Glen Rose Formation “the Glen Rose turtle,” Texas, USA, have been previously described, reported or included in phylogenetical studies (see Barck, 1992; Vineyard, 1999; Rogers, 2000; Vineyard, 2007; Vineyard, 2009). Despite all these studies, only Vineyard (2009) in her Master of Science thesis at the Southern Methodist University attributed the “Glen Rose turtle” to a new genus and species as “*Glenrosechelys brooksi*.” Following the fourth edition of the International Code of Zoological Nomenclature (ICZN, 1999) Article 8. “What constitutes published work” and considering that at present (after almost 6 years) “*Glenrosechelys brooksi*” has not been officially published and therefore not satisfying the criteria of the ICZN. I define here a new taxon name that groups “*Glenrosechelys brooksi*” and the new colombian sandownid turtle described here, supported in that there are no marked morphological differences between these two turtles to be considered as two separate taxa. Small variations in size and shape of some skull bones can be attributed to intraspecific ontogenetic variations, as Vineyard (2009) pointed out that occurs in “*Glenrosechelys brooksi*” skulls.

## Description

### Skull

The skull of *Leyvachelys cipadi* (FCG-CBP-71 specimen) is fairly complete, missing the most anterodorsal region, prefrontals, most of frontals, the dorsal portions of maxillae and premaxillae, and most of the ventral portions of jugals, quadratojugals at the cheek emargination region. No major deformation is apparent and the structures are tridimensionally preserved. In dorsal view (Figs. 2A and 2B), both parietals are expanded posteriorly and in lateral contact with postorbitals and squamosals to form a very reduced upper temporal emargination. The postorbitals are very long bones, in posterior contact with the squamosals and laterally with the jugals, the quadratojugals, and the maxillae. The squamosal covers most of the dorsal margin of the otic ring, which is formed by the quadrate. Both maxillae are very thick and tall bones. The premaxillae are preserved, however it is unclear if they are fused or medially sutured.

In the ventral view (Figs. 2C and 2D), the most anterior palatal region is covered by the articulated lower jaw. Pterygoids meet medially and are projected posteromedially as a concave wing completely covering the basisphenoid and the most anterior portion of the basioccipital. Laterally (Figs. 3A and 3B), the quadrate forms the C-shaped cavum tympani, being small and deep, with the incisura columellae auris enclosing the stapes via a long sutured contact. The articular processus of the quadrate is long and ends in a very wide mandibular condyle facet, posteriorly shifted. Anteriorly, the quadrate contributes together with the pterygoid to the very massive processus trochlearis oticum, better observed in ventrolateral view (Figs. 3C and 3D).

In the posterior view of the skull (Figs. 3E and 3F), a cervical vertebra is preseved, fragmented in two pieces, each of these located on the right and left side of the fossa temporalis superior respectively (see neck and tail for description). The exoccipitals form the condylus occipitalis and most of the lateral wall of the foramen magnum, however their sutural contacts with other bones are not clearly defined due to bad preservation of this area of the skull. A small portion of the crista supraoccipitalis is visible, however its posterior-most tip is broken. The opisthotics have a blade-like shape, very expanded laterally, in contact with squamosals and quadrates. A well deep canal is formed between the lateral projections of the pterygoids and the paroccipital process. At the basioccipital-pterygoid contact there is evidence of a groove for the foramen posterius canalis carotici interni, this groove is lateral to the tuberculum basioccipitale, which is well defined and very rounded.

### Lower jaw

The lower jaw of *Leyvachelys cipadi* is well preserved and articulated with the skull, missing the most posteroventral region of left ramus, including the mandibular condyle and some portions of the dentary, the surangular, and the angular. In ventral view (Figs. 2C and 2D), the dentary is fused forming a robust flat bony plate, very long at the symphysis. Posteriorly the dentary contacts the angular and surangular, and laterally the coronoid. The mandibular condyle is formed mostly by the articular, being wider than long. In lateral view (Figs. 3A and 3B), the coronoid is very tall and has an anteroventral contact with the dentary and posteroventral with the surangular. In dorsoventral view (Figs. 3C and 3D) the dentary exhibits a relatively deep depression, located anterolateral to the processus coronoideus.

### Neck and tail

**Figure 6:**
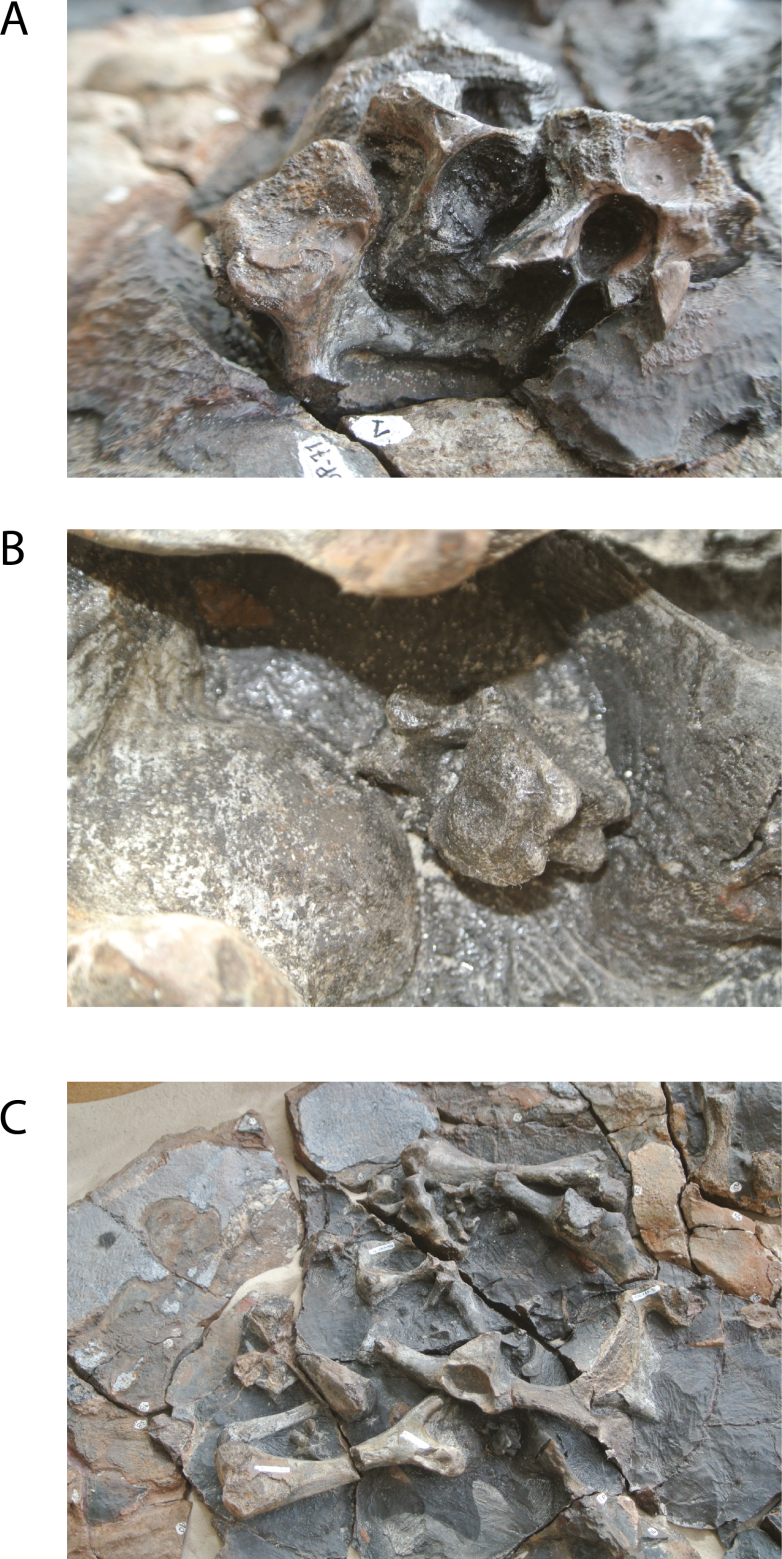
Photographs of *Leyvachelys cipadi* FCG-CBP-71. (A) cervical vertebrae 6 to 8, cervical 6 (right), cervical 7 (left), cervical 8 (middle back). (B) caudal vertebra located under left pubis. (C) dorsolateral view of the posterior region of the carapace, showing the pelvic girdles and hindlimb elements.

Four cervical and four caudal vertebrae of *Leyvachelys cipadi* are preserved. One cervical, potentially cervical 5?, is fragmented in two pieces, located at the left and right fossa temporalis superior of the skull (Figs. 3E and 3F). The other three cervicals, potentially 6? to 8 are preserved on the ventral surface of nuchal bone, being all procoelous. Cervical 8 is almost located at its anatomical position, close to dorsal vertebrae 1, and slightly shorter than the other (Figs. 4C and 4D). The cervicals of *L. cipadi* have a very height neural spine (better preserved in cervical 5?), slightly shorter in posterior cervicals (6? to 8) (Fig. 6). Cervicals 5 and 6 are preserved in anterior view, both having prezygapophyses facing dorsolaterally and concave, having slightly elongated centra. Cervical ribs are absent. The transverse process of cervicals is ventrolaterally projected and located at the anterior end of the cervical centrum. Cervicals of *L. cipadi* also have a short ventral process (keel) ending in acute tip, preserved in cervical 5? and 6?, unknown for the other two cervicals. The anterior centrum of cervical 8 (Fig. 6A) is also concave, oval in shape and without evidence of a biconvex condyle articulation with cervical 7. The four preserved caudal vertebrae of *L. cipadi* (Figs. 4E and 4F) are located one besides the right femur, one close to the thoracic vertebra 10, one besides the left femur, and one slightly under the left pubis (Fig. 6B). Three of the four caudals exhibit amphicoelous central articulations, with a very short to absent neural spine and long transverse processes. On the other hand, one of the caudals shows a slightly convex central articulation indicating that is procoelous with very reduced prezygapophyses, and ventral medial notch, instead of keels, potentially an anterior caudal.

### Limbs

The complete right humerus, both femora, portions of the right tibia and fibula, and an isolated metacarpal are preserved for *Leyvachelys cipadi* (Figs. 4E and 4F). The humerus has a nearly spherical head with a very narrow and short lateral process. The medial process is thick and longer than the lateral, with a very rounded dorsal surface. The shaft of the humerus in *L. cipadi* has a sigmoidal curve, being almost circular in cross section at its narrower region. The ectepicondylar foramen is surrounded by bone emerging on the ventral surface just anterior to the ulnar articulation margin. Both femora are preserved in dorsal view and they are longer than the femur (See Table 1 for measurements). The femoral head is spherical and located between the trochanters minor and major, which are laterally short with rounded dorsal surface, both located at the same horizontal level. The femur of *L. cipadi* has very robust tibial articulation, and as the humerus the shaft is circular in cross section at its narrower level, not developing any major flattening of the general morphology of the bone. The isolated metacarpal is short and lacks of any longitudinal flattening, with distal articulations circular in shape seen in lateral view. (Fig. 4F).

### Pectoral and pelvic girdles

The right coracoid and partial scapula from the pectoral girdle (Figs. 4E and 4F) are preserved but disarticulated, and both pelvic girdles of *Leyvachelys cipadi* are also preserved, completely articulated, but slightly moved towards right from their anatomical position, missing the most ventral portions of ischia and the left lateral pubic process (Figs. 4E and 4F, Fig. 6C). The coracoid is shorter than half of the scapula and it has a narrow neck close to the glenoid, with distal blade rounded and broad. The half portion of the scapula is narrow and long stick-like shape bone. The pelvis exhibit a medial sutural contact between pubes, well defined and roughly square lateral pubic process, and an the acetabulum capsule is oval elongated in shape, having the sutural contact between pubis, ischium, and ilium. The ilia are almost parallel to each other, long and narrow, having a short ventrodistal process. The ischia are not preserved, however their base at the acetabulum indicates the possible existence of large thyroid fenestra.

### Carapace

The carapace of *Leyvachelys cipadi* is nearly completely preserved, with a series of ten dorsal vertebrae, only missing some portions of the costal bones (Figs. 4A–4D). The carapace is slightly wider than long, however this could be as a result of crushing. Due to bad preservation of the bone surface (dorsal and ventral) particularly by a thin layer of oxides and the development of siderite nodules, sutures and sulci are poorly preserved for most of the carapace, except by some sutural contacts between peripherals and costals, which are strongly ossified and lacking of fontanelles or reduction in bone thickness. Peripherals (1 to 4) are rectangular slightly wider than long; bridge peripherals (5 to 7) are almost square in shape, showing on their ventral surface the sutural scar for the hyoplastron and hypoplastron articulation, which is continuous, indicating that the plastron (which is not preserved), lacked of large lateral fontanelles at the bridge region. The most posterior peripherals (8 to 11) and pygal are slightly longer than wide, and larger in size compared to the anterior and bridge peripherals. There is not evidence of axillary scar reaching costals, however that is not the case for the inguinal scar, which slightly touches costal 5?. Dorsal vertebrae are hourglass shape in ventral view, 1 to 5 having a wider contact between them compared to the posteriors 6 to 10, for which the contact is narrower. Dorsal vertebra 8 to 10 form the sacral vertebral series, with left sacral rib 9 and 10 clearly defined.

On the dorsal surface of the carapace, only neurals 1 and 2 are barely identifiable, any other suture or bones outline cannot be undisputable outlined. On one of the posterior left costal bones, there is evidence of a sulcus possibly between vertebral scute 4 and 5. There is not evidence of a clear sculpturing pattern, neither pits, striations or vermiculations.

## Discussion

### Phylogenetical considerations

**Figure 7:**
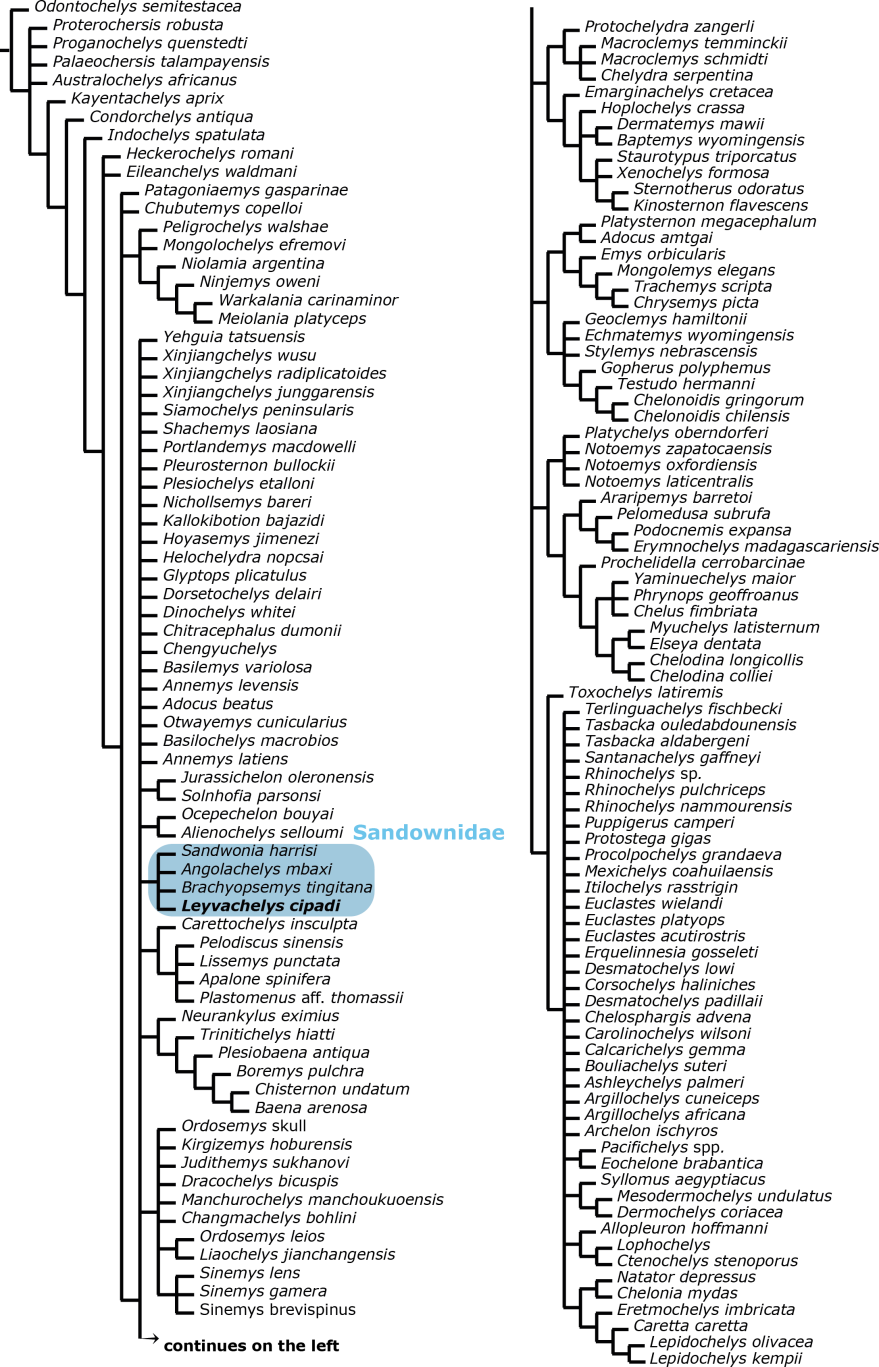
Global Testudines phylogenetic tree. Strict consensus tree from 96 most parsimonious trees, showing the place of Sandownidae clade (blue shaded rectangle) among a global turtles phylogeny. All taxa and all characters included (see Supplemental Information 1 and Supplemental Information 2, and first analysis on methods).

**Figure 8:**
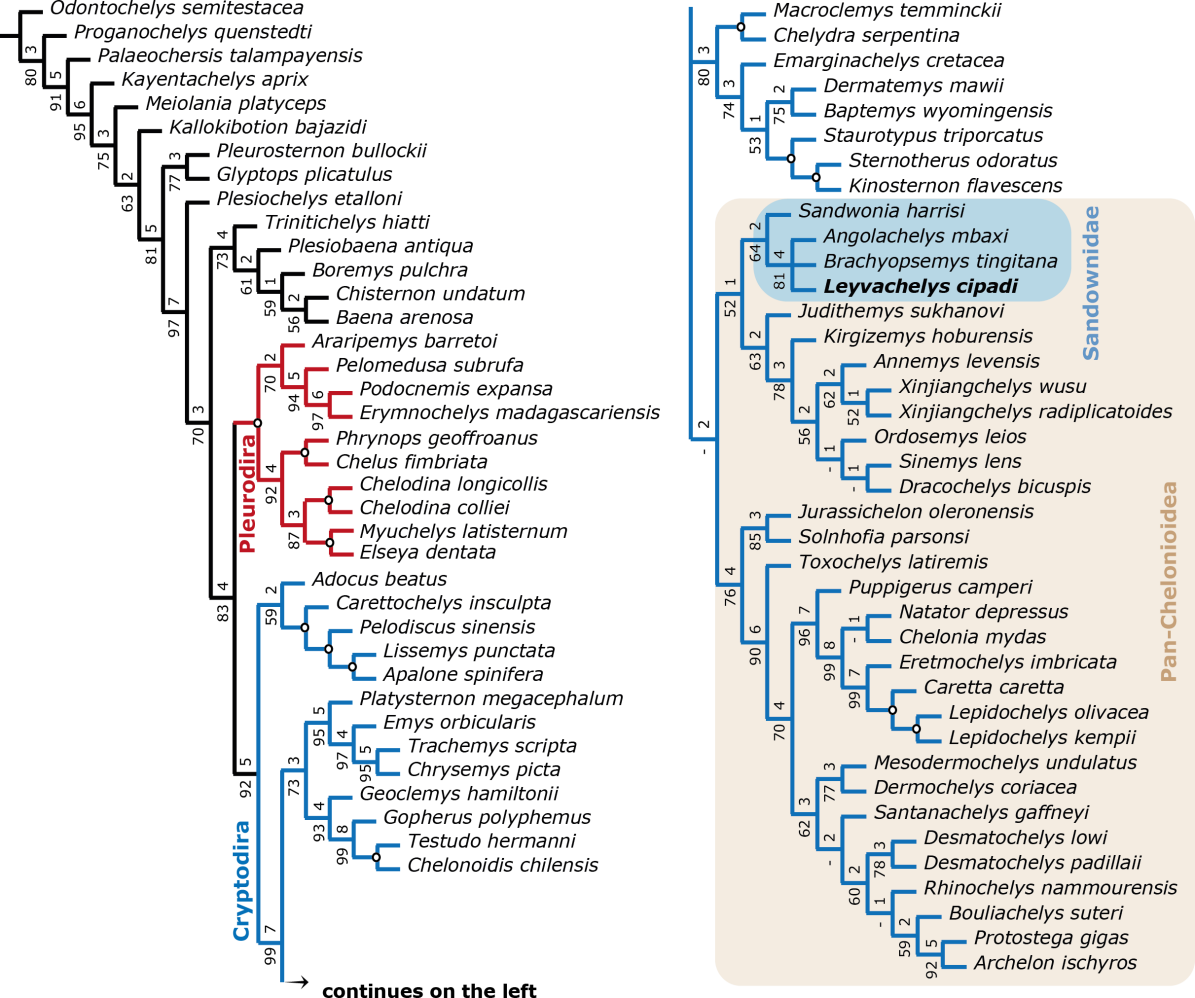
Filtered phylogenetic tree of Testudines. (A) Strict consensus of 3 most parsimonious trees (see ‘Methods’, second analysis). Including all taxa used in Cadena & Parham (2015, Fig. 11) after removing wildcard taxa and taxa with high percentage of missing data, with the addition of the four sandownids. Pleurodira turtles are represented by red lines and Cryptodira by blue lines. Bootstrap support values from 100 replicates are shown below and Bremer decay indices above each node. Nodes with bootstrap values of 100 and Bremer indices of 9 or more are shown with an open circle. Pan-Cheloniodea clade is delimitated by the brown shaded rectangle.

A strict consensus tree from 96 most parsimonious trees was found from the global phylogenetic analysis including all taxa and all characters (Fig. 7). Tree length (TL:1211), consistency index (CI:0,602) retention index (RI:0,495). The general topology of this tree shows a very large polytomy including stem and crown-Testudines. In spite of this large polytomy, sandownids are resolved as monophyletic and not included in the large Chelonioidea *sensu lato* clade following Cadena & Parham (2015). With the aim to obtain a better understanding of the phylogenetic relationship of sandownids among Testudines, a second analysis was run (see ‘Materials & Methods’), obtaining 3 most parsimonious trees (see Fig. 8 for the strict consensus) TL:949, CI:0.778, RI:0.325. Once again Sandownidae is found as monophyletic clade, however this time being part of Pan-Chelonioidea and close related to the clade form by *Judithemys*-xingjianchelyids-sinemydids. However I point out here that the obtained boostrap values and Bremer support indexes found for the Sandownidae clade as well as to support the inclusion of *Judithemys*-xingjianchelyids-sinemydids as pan-chelonioids are low, requiring only one or two steps for the clades to collapse within Cryptodira. The phylogenetical position of Sandownidae as part of Pan-Chelonioidea found here supports the hypotheses of Tong & Meylan (2013); however, it is important to point out that the Pan-Chelonioidea clade defined on here is not exclusive of open marine turtles, and also includes freshwater Jurassic and Cretaceous turtles (macrobaenids, sinemydids, and xinjiangchelyids), which are still being problematic and unstable in many phylogenetical studies (see discussion in Cadena & Parham, 2015). Once again, as Tong & Meylan (2013) found using a relatively different character dataset, the Jurassic turtle *Solnhofia parsoni* is excluded from Sandownidae clade, although found to be a part of Pan-Chelonioidea. Inside Sandownidae clade, the relationships between taxa are also still unresolved; however, as *Sandownia harrisi* is placed out of the polytomy formed the other three members of the clade, this can be attributed in part to the lack of synapomorphies, as well as high percentage of missing data in the character-taxon matrix.

### Sandownids versus other litoral and open marine turtles

Morphological comparisons between the skull and jaws of sandownids have been discussed in Mateus et al. (2009) and Tong & Meylan (2013: Table 13.2) including the unnamed Glen Rose turtle, attributed here to *Leyvachelys cipadi*. Instead of being repetitive with the comparisons between sandownids, I dedicate this session to discuss new or missing important morphological characteristics not previously considered in the literature, as well as to compare the sandownids with other turtle lineages, particularly with those that show similar morphological developments, as for example the secondary palate or the pterygoid posteromedial wing.

**Figure 9:**
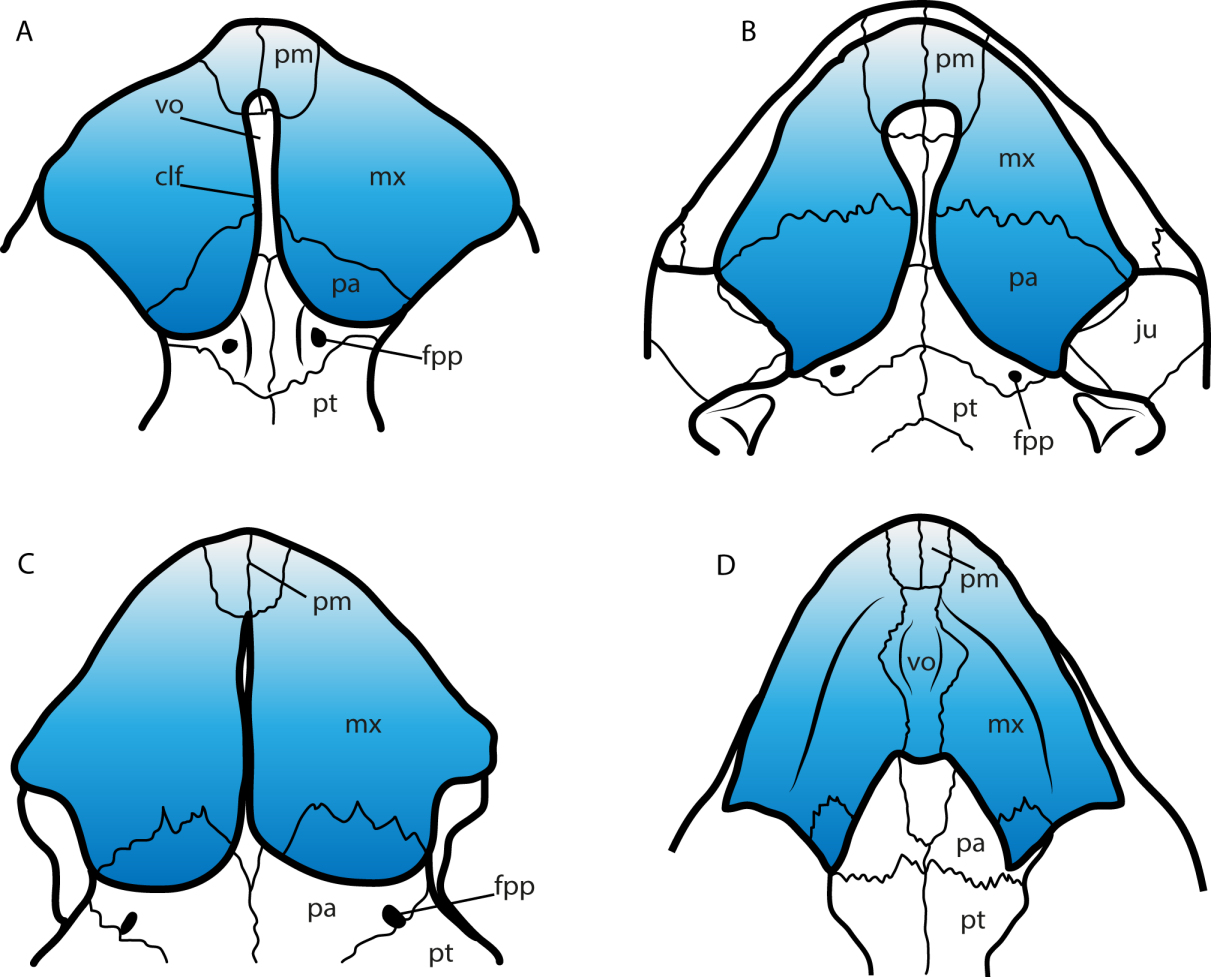
Secondary palate configuration for four different groups of turtles. (A) *Leyvachelys cipadi* SMU 75355 (Sandownidae), redrawn from Vineyard (2009). (B) *Bairdemys veenezuelensis* (Podocnemididae), redrawn from Gaffney & Wood (2002). (C) *Emydura victoriae* MTKD (Senckenberg Museum, Herpetological Collection, Dresden) 42435 (Chelidae). (D) *Chelonia mydas* MTKD 3939 (Cheloniidae). Abbreviations: clf, cleft; fpp, foramen palatinum posterious; ju, jugal; mx, maxilla; pa, palatine; pm, premaxilla; pt, pterygoid; vo, vomer.

A feature of the turtle palate independently acquired by different groups is the secondary palate. Defined by the bony separation of the narial cavity from the oral cavity (Parham & Pyenson, 2010) forming two separate plates, which in most of the cases is accompanied by a wide expansion of the triturating surface, specifically useful for turtles having a durophagous diet (Parham & Pyenson, 2010; Ferreira et al., 2015, and references therein). Secondary palate, although with differences in the configuration of bones and extension (Fig. 9), is present in almost all cheloniids (cryptodires, marine adapted) (see Parham & Pyenson, 2010), some geoemydids and some emydids (both cryptodires, freshwater adapted) (see Claude et al., 2004), some bothremydids (pleurodires, costal-littoral adapted) (see Gaffney, Tong & Meylan, 2006), some podocnemidids (pleurodires, costal-littoral adapted) (see Ferreira et al., 2015), in the chelid *Emydura victoriae* Gray, 1841 (pleurodire, freshwater adapted) (examined specimen MTKD 42435), and also in the sandownids (eucryptodires, costal-littoral adapted) (see Tong & Meylan, 2013 and this study). The secondary palate of *Leyvachelys cipadi* and *Sandownia harrisi* exhibit a narrow midline cleft (Fig. 9A), similar to the stereogenyins, i.e., *Bairdemys venezuelensis* Gaffney & Wood, 2002 (Fig. 9B) and the chelid *E. victoria* (Fig. 9C). However, in the case of the two sandownids, the midline cleft has the contribution of the vomer in its formation. In *Angolachelys mbaxi* and *Brachyopsemys tingitana*, the midline cleft is extremely reduced or absent, as in cheloniids, e.g., *Chelonia mydas* (Fig. 9D).

Another important morphological feature of the skull of sandownids is the development of a pterygoid posteromedial wing completely covering the basisphenoid and the most anterior portion of basioccipital, accompanied also by a large posterolateral extension of pterygoids. The degree of covering of the basisphenoid is suggested to increases during ontogeny (Vineyard, 2009). Other turtles have also developed posterolateral extension of pterygoids, as for example trionychids; however, their pterygoids lack of posteromedial expansion and also exhibit medial contact between them.

The lower jaw of *L. cipadi* and *B. tingitana* exhibit also some similarities with some cryptodires, as for example *Erquelinnesia gosseleti* and ? *Palatobaena* sp. (Figured in Gaffney, 1979: Figs. 161 and 196, respectively), and the Jurassic *Solnhofia parsonsi* in having a deep concave depression of the dentary and coronoid, located anterolateral to the processus coronoideus, which corresponds in extant cheloniids with the area for the insertion of the M. Adductor Mandibulae Externus, particularly the superficialis (No. 21C) (Jones et al., 2012). This suggests that probably sandownids had similar musculatory articulations between skull and jaw as those of some extant cheloniids, which feed on relatively slow moving but sometimes armoured prey (Jones et al., 2012), indicating also that these morphological features were acquired independently by this two groups of turtles.

One of the most relevant morphological features of *Leyvachelys cipadi* (FCG-CBP-71 specimen) is the preservation of the limbs, pectoral, and pelvic elements. The humerus of *L. cipadi* is shorter than the femur, longer than the coracoid, lacks a distally shifted lateral process. (Parham & Pyenson, 2010; Cadena & Parham, 2015). Also, the humerus and femora of *L. cipadi* lack of flattening and widening of distal regions, as it is the condition in chelonioids (Hirayama, 1997: Fig. 8). In all these aspects, the limbs of *L. cipadi* are more similar to the ones from freshwater or costal-littoral turtles than of those highly marine adapted turtles. For example, the humerus of *L. cipadi* ressembles the humerus of the stem cryptodire *Osteopygis (sensu* Parham, 2005) (Figured in Hirayama, 1997: Fig. 8A) and some freshwater cryptodires as for example *Macroclemys temmincki* (Figured in Gaffney, 1990: Fig. 149C). This is also the case of the isolated metacarpal of *L. cipadi* which differs from the flatter and longer metacarpals of chelonioids (see Joyce, 2007: Fig. 14). The pelvic girdle of *L. cipadi* also resembles the pelvis of extant freshwater cryptodires, with a long medial contact between pubes, a short but robust lateral pubic process, and a columnar ilium (seen in ventral view) (see Gaffney, 1990: Fig. 144C).

The carapace of *L. cipadi* is the first complete ever found for sandownids, showing a strongly ossified sutural contact between peripherals and costals, lacking fontanelles or naked ribs of costals, which are present in fully adapted marine turtles as for example protostegids and cheloniids, during all their ontogenetic stages (see Hirayama, 1997: Figs. 2–6, Cadena & Parham, 2015). Besides, *L. cipadi* carapace exhibits a very strong and continuous plastral bridge scar similar for example as the carapace of the stem cryptodire *Ostepygis* (Parham, 2005: Fig. 4A). This is different from the condition of chelonioids, which have a large lateral fontanella between hyo-hypoplastra, or with just a weak, nearly touching sutural contact between plastron and carapace (see Hirayama, 1997: Figs. 2–6). Furthermore, the few well-preserved dorsal surface areas of the carapace of *L. cipadi* are smooth, lack of any particular sculpturing pattern, differing from trionychians, a group to which sandownids were initially attributed (Meylan et al., 2000). Smooth dorsal surface of carapace is also the condition reported for undescribed and not figured shell fragments of *Angolachelys mbaxi* (see Mateus et al., 2009), indicating that it was possibly the general condition in all sandownids.

### Paleoecology and paleobiogeography of sandownids

The morphology of the shell of *Leyvachelys cipadi* (FCG-CBP-71 specimen), allows the support of previously hypothesized habitat adaptations for sandownids; in particular, that they inhabited littoral to near-shore shallow marine environments Tong & Meylan (2013), and that their general body-plan was not designed for leading an open marine lifestyle. They nevertheless potentially shared niches with open marine turtles, as evidenced by the occurrence of protostegids from the same stratigraphical horizons (Cadena & Parham, 2015). The abundant occurrence of mollusks, principally ammonites, some of them preserved associated with the carapace of *L. cipadi*, suppose a potential source of food for its durophagous diet adaptation which could have also included artropods, as for example crabs.

**Figure 10:**
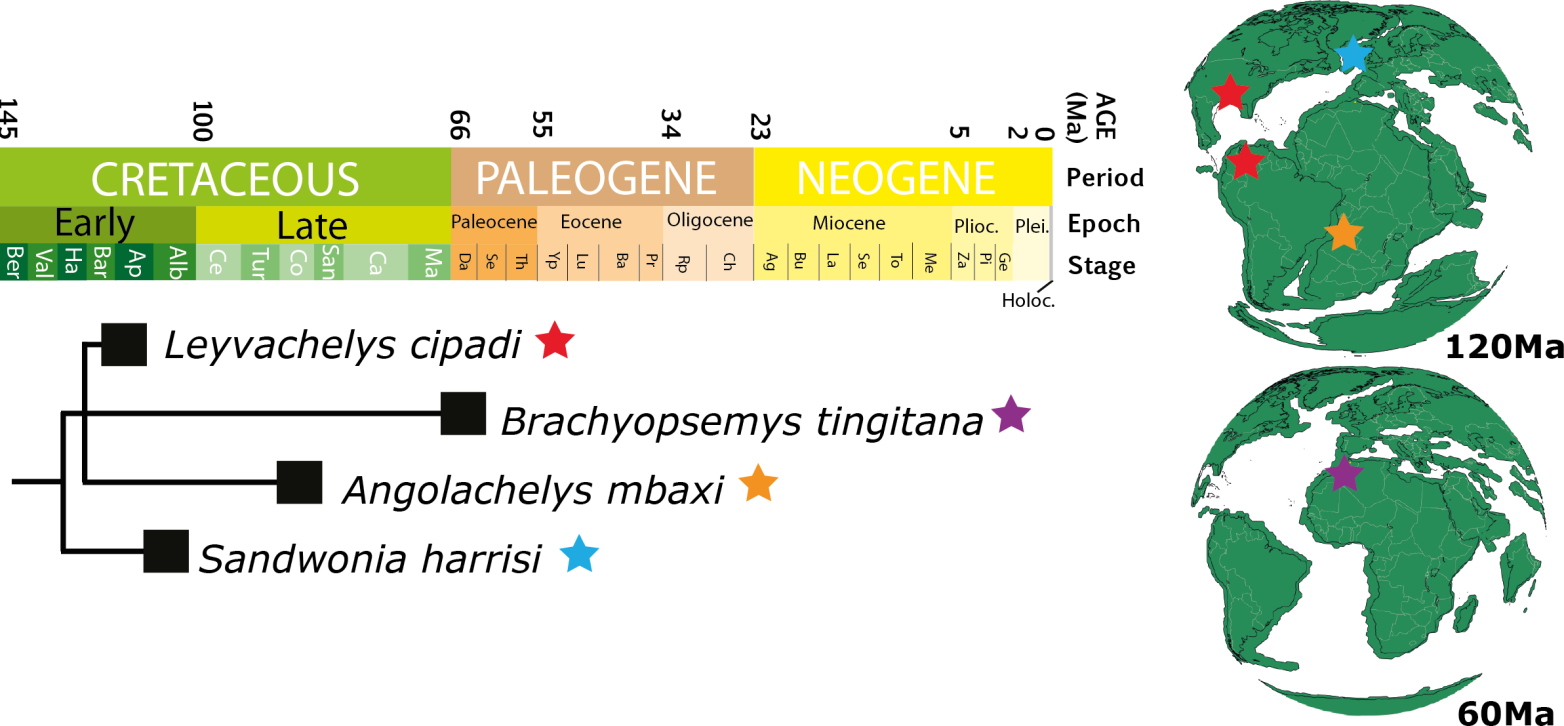
Chronostratigraphic distribution of sandownids. Matching the topology presented in Fig. 8 (this study), and their geographical distribution for the Cretaceous (upper globe) and for the Paleogene (lower globe). Tectonic reconstructions at 120 Ma and 60 Ma were created using the analyze tools on *fossilworks.org.*

*Leyvachelys cipadi* not only expands back to the upper Barremian-lower Aptian (>120 Ma) the fossil record of sandownids, but also expands their paleogeographical distribution, being the first record of sandownids in South America. Paleotectonic reconstructions for the Barremian-Aptian of the Gulf of Mexico and the porto-caribbean (Pindell & Kennan, 2009; Blakey, 2011) (Figs. 1E and 10), suggest the existence of an almost continuous littoral areas between the Gulf of Mexico and northern South America, which could have served as a corridors for the dispersion or migrations of marine-littoral vertebrates including *Leyvachelys cipadi* giving explanation to its occurrence in Glen Rose Formation of Texas and Paja Formation of Colombia. As mentioned by Tong & Meylan (2013), the evolutionary history and dispersion of sandownids (now with a Barremian to Paleocene stratigraphic range and a geographical distribution including South America, North America, Europe, and Africa) was influenced by the opening of the Atlantic Ocean. This seems to be also the case for other groups of littoral to costal turtles, as for example the bothermydid pleurodires (Gaffney, Tong & Meylan, 2006; Cadena, Bloch & Jaramillo, 2012).

## Supplemental Information

10.7717/peerj.1431/supp-1 Supplemental Information 1 Character added to the character list of Cadena & Parham (2015), as well as changes in scoring.

10.7717/peerj.1431/supp-2 Supplemental Information 2 Character-Taxon matrix (Nexus file) used for the phylogenetical analysis.

## Additional Information and Declarations

### Data Availability

The following information was supplied regarding data availability: The research in this article did not generate any raw data.

### New Species Registration

The following information was supplied regarding the registration of a newly described species: urn:lsid:zoobank.org:pub:5ADF956A-ADA5-4D72-9DFD-2ABE923366E4.
