# Supplementary material for: The first South American sandownid turtle from the Lower Cretaceous of Colombia
Source: PeerJ. 2015 Dec 15;3:e1431. doi: 10.7717/peerj.1431 (PMC4690369; doi:10.7717/peerj.1431)
Supplement: Supplemental Information 1 — Character added to the character list of Cadena & Parham (2015), as well as changes in scoring. [file peerj-03-1431-s001.docx]

**Supplemental Information 1**

**Character added to the character list of *Cadena & Parham. (2015)***

**257.** Pterygoids, development of a posteromedial wing covering partially to completely the basisphenoid and sometime the basioccipital too, seen in ventral view of the skull: 0 = absent; 1 = present. (**Tong & Meylan, 2013, ch 148**). *Remarks*: character redefined in terms of the pterygoid wing, which regulates the ventral exposure of the basisphenoid and basioccipital.

**Changes in scoring on the *Cadena & Parham, 2015* character-taxon matrix**

Character 40

*Brachyopsemys tingitana* from 0 to 1

*Angolachelys mbaxi* from 0 to 1

Character 41

*Brachyopsemys tingitana* from 0 to 1

*Angolachelys mbaxi* from 0 to 1

*Sandwonia harris* from 0 to 1

Character 42

*Sandwonia harris* from 1 to 0

Character 51

*Sandwonia harris* from 1 to 0

Character 57

*Angolachelys mbaxi* from 1 to 2

Character 62

*Angolachelys mbaxi*  from 0 to 1

Character 66

*Sandwonia harris* from 2 to 0

Character 67

*Angolachelys mbaxi* from 1 to 0

*Sandwonia harris* from 2 to 0

Character 68

*Brachyopsemys tingitana* from 1 to 0

*Sandwonia harris* from ? to 1

Character 72

*Sandwonia harris* from 0 to 2

Character 80

*Brachyopsemys tingitana* from 0 to -

*Angolachelys mbaxi* from 0 to -

*Sandwonia harris* from 0 to -

Character 86

*Sandwonia harris* from 1 to -

Character 87

*Brachyopsemys tingitana*  from 0 to -

*Angolachelys mbaxi*  from 0 to -

*Sandwonia harris*  from 0 to -

Character 88

*Brachyopsemys tingitana*  from 0 to -

*Angolachelys mbaxi*  from 0 to -

*Sandwonia harris*  from 0 to -

Character 89

*Brachyopsemys tingitana*  from 0 to -

*Angolachelys mbaxi*  from 0 to -

*Sandwonia harris*  from 0 to -

Character 110

*Sandwonia harris* from 0 to 1

**Taxa considered as floaters during the phylogenetic analysis.**

*Annemys latiens*

*Basilochelys macrobios*

*Changmachelys bohlini*

*Liaochelys jianchangensis*

*Manchurochelys manchoukuoensis*

*Otwayemys cunicularius*

*Plastomenus aff. thomassii*

*Sinemys brevispinus*

*Stylemys nebrascensis*

*Xenochelys formosa*

*Adocus amtgai*

*Adocus beatus*

*Alienochelys selloumi*

*Allopleuron hoffmanni*

*Annemys levensis*

*Araripemys barretoi*

*Archelon ischyros*

*Argillochelys africana*

*Argillochelys cuneiceps*

*Ashleychelys palmeri*

*Baptemys wyomingensis*

*Basilemys variolosa*

*Boremys pulchra*

*Bouliachelys suteri*

*Calcarichelys gemma*

*Carolinochelys wilsoni*

*Chelonoidis gringorum*

*Chelosphargis advena*

*Chengyuchelys*

*Chisternon undatum*

*Chitracephalus dumonii*

*Desmatochelys padillaii*

*Corsochelys haliniches*

*Ctenochelys stenoporus*

*Desmatochelys lowi*

*Dracochelys bicuspis*

*Echmatemys wyomingensis*

*Emarginachelys cretacea*

*Eochelone brabantica*

*Erquelinnesia gosseleti*

*Euclastes acutirostris*

*Euclastes platyops*

*Euclastes wielandi*

*Helochelydra nopcsai*

*Hoplochelys crassa*

*Hoyasemys jimenezi*

*Itilochelys rasstrigin*

*Judithemys sukhanovi*

*Kirgizemys hoburensis*

*Lophochelys*

*Macroclemys schmidti*

*Mesodermochelys undulatus*

*Mexichelys coahuilaensis*

*Mongolemys elegans*

*Nichollsemys bareri*

*Niolamia argentina*

*Notoemys laticentralis*

*Notoemys oxfordiensis*

*Notoemys zapatocaensis*

*Ocepechelon bouyai*

*Ordosemys leios*

*Ordosemys* skull

*Pacifichelys* spp*.*

*Platychelys oberndorferi*

*Plesiochelys etalloni*

*Portlandemys macdowelli*

*Prochelidella cerrobarcinae*

*Procolpochelys grandaeva*

*Protochelydra zangerli*

*Protostega gigas*

*Puppigerus camperi*

*Rhinochelys nammourensis*

*Rhinochelys pulchriceps*

*Rhinochelys* sp*.*

*Santanachelys gaffneyi*

*Shachemys laosiana*

*Siamochelys peninsularis*

*Sinemys gamera*

*Sinemys lens*

*Solnhofia parsonsi*

*Syllomus aegyptiacus*

*Tasbacka aldabergeni*

*Tasbacka ouledabdounensis*

*Terlinguachelys fischbecki*

*Jurassichelon oleronensis*

*Toxochelys latiremis*

*Xinjiangchelys junggarensis*

*Xinjiangchelys radiplicatoides*

*Xinjiangchelys wusu*

*Yaminuechelys maior*

*Yehguia tatsuensis*

*Leyvachelys cipadi*

*Brachyopsemys tingitana*

*Angolachelys mbaxi*

*Sandwonia harrisi*
